# New Dihydro OO′Bis(Salicylidene) 2,2′ Aminobenzothiazolyl Borate Complexes: Kinetic and Voltammetric Studies of Dimethyltin Copper Complex with Guanine, Adenine, and Calf Thymus DNA

**DOI:** 10.1155/BCA/2006/32896

**Published:** 2006-03-05

**Authors:** Farukh Arjmand, Bhawana Mohani, Shamima Parveen

**Affiliations:** Department of Chemistry, Faculty of Science, Aligarh Muslim University, Aligarh 202 002, India

## Abstract

The newly synthesized ligand, dihydro OO′bis(salicylidene) 2,2′ aminobenzothiazolyl borate (2), was derived from the reaction of Schiff base of 2-aminobenzothiazole and salicylaldehyde with KBH_4_. Cu^II^ (3) and Zn^II^ (4) complexes of (2) were synthesized and further metallated with dimethyltindichloride to yield heterobimetallic complexes (5) and (6). All complexes have been thoroughly characterized by elemental analysis, and IR, NMR, EPR, and UV-Vis spectroscopy and conductance measurements. The spectroscopic data support square planar environment around the Cu^II^ atom, while the Sn^IV^ atom acquires pentacoordinate geometry. The interaction of complex (5) with guanine, adenine, and calf thymus DNA was studied by spectrophotometric, electrochemical, and kinetic methods. The absorption spectra of complex (5) exhibit a remarkable “hyperchromic effect” in the presence of guanine and calf thymus DNA. Indicative of strong binding of the complex to calf thymus DNA preferentially binds through N_7_ position of guanine base, while the adenine shows binding to a lesser extent. The kinetic data were obtained from the rate constants, *k_obs_*, values under pseudo-first-order conditions. Cyclic voltammetry was employed to study the interaction of complex (5) with guanine, adenine, and calf thymus DNA. The CV of complex (5) in the absence and in the presence of guanine and calf thymus DNA altered drastically, with a positive shift in formal peak potential *E_pa_* and *E_pc_* values and a significant increase in peak current. The positive shift in formal potentials with increase in peak current favours strong interaction of complex (5) with calf thymus DNA. The net shift in *E*_1/2_ has been used to estimate the ratio of equilibrium constants for the binding of Cu(II) and Cu(I) complexes to calf thymus DNA.

## INTRODUCTION

Present-day anticancer agents are facing challenges such as side effects, toxicity, targeting, drug delivery, acquired resistance, and cancer specificity. To overcome such problems, drugs with different molecular level action are required, and a variety of such species are under way to address these problems [1–4]. The interaction of drugs at the target site involves the DNA helix which is a sequence of bases (thymine, adenine, guanine, and cytosine). The correct conformation of the DNA helix is maintained only when adenine is paired with thymine and guanine with cytosine. A large number of articles appearing in the literature describe the interaction of metal ions with nucleic acids, giving the mechanism of action of metal-based chemotherapeutic agents that target DNA [5–10].

Developing new rational designer antitumor drugs on similar mechanisms of cisplatin, which target the cellular DNA and exhibit chemical similarity of N_7_ binding site in guanine and adenine, is a most challenging area in the pharmaceutical industry. In a recent article, Lippard et al studied the theoretical binding of cisplatin to purine bases; a dominating preference for initial attack at N_7_-position of guanine in comparison with adenine has been established [11]. On the basis of Δ*G* ^‡^, the activation free energy values for guanine = 24.6 kcal/mol and adenine 30.2 kcal/mol, it was predicted that guanine is 3-4 orders of magnitude more reactive towards cisplatin than adenine.

Boron compounds have received considerable attention as biologically important molecules, since boron is an essential element and is involved in nucleic acid synthesis linked to pyrimidine nucleotides [12]. They may be utilized to probe fundamental biochemical events at the molecular level as well as in providing entirely new classes of compounds of potential medicinal value. Based upon four-coordinate boron, they generally possess sufficient hydrolytic and oxidative stability to be used in biological studies [13, 14]. The development of boron compounds for the treatment of cancer by boron neutron capture therapy (BNCT) is very significant. The closer the proximity of boron compound to tumor cell nucleus, the greater its radiobiological effect [14]. Many researchers are studying boron-containing molecules as potential delivery agents for cancer chemotherapy [15].

Boron compounds in combination with tin(IV) metal cation (hard Lewis acid), which exhibits strong affinity to the dinegative phosphate groups of DNA [16] (supportive evidence for this coordination exists both in solution [17] and in solid state [18] and tin compounds are reported to be effective against some types of cancers, such as P-338 leukemia [19, 20]), have further advantage of exhibiting therapeutic success by healing the damaged cells.

Herein, we describe the kinetics and electrochemical behavior of the representative complex (5) towards guanine, adenine, and calf thymus DNA to understand the mechanistic pathway of binding to cellular targets. These studies were carried out using UV-Vis spectroscopy and cyclic voltammetry mainly 1. The binding ability of the complex is multifold due to the presence of these metal ions which selectively bind to the target site viz copper. A transition metal ion prefers to bind to N_7_ of guanine or adenine to a lesser extent of the nucleotide bases, while tin(IV) cation binds to the phosphate group of the DNA backbone [21], and boron atom provides possible cellular entrapment and retention properties in proliferating tumor cells [14].

## EXPERIMENTAL

### Materials and methods

All the reagents 2-aminobenzothiazol (Farak berlin, Germany), salicylaldehyde, KBH_4_ (Lancaster), Calf thymus DNA, guanine, adenine (Sigma), (CH_3_)_2_SnCl_2_ (Fluka), CuCl_2_ · 2H_2_O, and ZnCl_2_ (anhydrous) (Merck) were used without further purification. Microanalyses were performed by a Carlo Erba Analyzer Model 1108. Molar conductance was determined at room temperature by a Digisun electronic conductivity bridge. IR spectra (Nujol mull) (200−4000 cm^−1^) were recorded on a Shimadzu 8201 PC spectrophotometer. ^1^H and ^13^C NMR spectra were recorded by Bruker DRX-300 spectrometer. Mass spectra were obtained on a Jeol SX-102 (FAB) spectrometer. EPR spectra were recorded on a Varian E112 spectrometer at X-band frequency (9.1 GHz) at liquid nitrogen temperature (LNT).

Cyclic voltammetry was carried out at CH instrument electrochemical analyzer. High purity H_2_O and DMSO (95 : 5) was employed for the cyclic voltammetric studies with 0.4 M KNO_3_ as a supporting electrolyte. A three electrode configuration was used comprising of a Pt disk working electrode, Pt wire counter electrode, and Ag/AgCl as reference electrode. Kinetic studies were carried out with a Cintra 5 UV-Vis spectrometer attached to an online data analyzer on which absorption spectra were evaluated. All experiments involving the interaction of the complex (5) with guanine, adenine, and calf thymus DNA were conducted in buffer (9.2 pH), doubly distilled water, and Tris buffer (7.5 pH), respectively. The progress of the reaction was monitored by measuring absorbance changes at 269 nm (λ_max_ of complex (5) + guanine), 260 nm (λ_max_ of adenine), and 260 nm (λ_max_ of CT-DNA), respectively. Pseudo-first-order rate constants, *k*_obs_, were determined by linear least squares regression method.

### Synthesis of Schiff base ligand (1)

To a solution of 2-aminobenzothiazol (5 g, 0.033 mol) in 50 mL of methanol was added (3.49 g, 0.033 mol) salicylaldehyde. The reaction mixture was refluxed for 3 hours. Yellow precipitate appears immediately on cooling, which was separated by filtration, recrystallized from methanol, and dried in vacuo over fused CaCl_2_. Yield 7.0 g (82%) mp 120 ± 2°C (found: C, 66.16; H, 3.90; N, 10.98. C_14_H_10_N_2_SO%) requires C, 66.14; H, 3.93; N, 11.02. IR/cm^−1^ (Nujol mull): 1608_vs_ (C=N), 1286 (C−OH), 753(C−S). *δ*_H_ (300 MHz, DMSO, TMS) 7.57-6.55 (ArH), 7.90-7.82 (HC=N), 10.12 (OH). *δ*_*c*_ 129-124 (ArC), 165 (HC=N), 152 (C−S).

### Synthesis of the dihydro OO′bis(salicylidene)2,2′ aminobenzothiazolyl borate (2)

To a solution of Schiff base (4.7 g, 0.018 mol) in 100 mL dry DMF was added KBH_4_ (0.5 g, 0.009 mol). This reaction mixture was refluxed for circa. 10 hours in a closed assembly fitted to monitor the evolution of H_2_ gas. During the course of refluxing the reaction mixture, the solution changes colour slowly from dark yellow to colorless and later turns brown. After 10 hours, the evolution of hydrogen gas ceased and light brown colored product appeared in the reaction flask. The product was filtered and washed twice with toluene and dried in vacuo over fused CaCl_2_. Yield 3.6 g (69%) mp 260 ± 1°C (found: C, 64.88; H, 3.81; N, 10.78. C_28_H_20_N_4_S_2_O_2_B requires C, 64.86; H, 3.86; N, 10.81%). Mass spectrum (FAB^+^): m/z 518. IR/cm^−1^ (Nujol mull): 1595_vs_ (C=N), 1391 (B−O), 2395 (B−H). ∧_M_ (CH_3_OH): 102 Ω^−1^ cm^2^ mol^−1^ (1 : 1 electrolyte). *δ*_H_ (300 MHz, DMSO, TMS) 7.55-6.52 (ArH), 7.99-7.97 (HC=N), 0.72 (B−H). *δ*_c_ 129-124 (ArC), 165 (HC=N), 114 (C−N), 153 (C−S).

### Synthesis of [C_28_H_22_N_4_S_2_O_3_BCuCl] (3)

The borate ligand (1.03 g, 0.002 mol) in 50 mL methanol was treated with CuCl_2_· 2H_2_O (0.340 g, 0.002 mol). The reaction mixture was stirred for 2 hours and then allowed to stand overnight in refrigerator. A brown product separates out, which was isolated by filtration under vacuum. It was washed thoroughly with hexane and dried in vacuo over fused CaCl_2_. Yield 0.57 g (54%) mp 205 ± 3°C (found: C, 53.00; H, 3.46; N, 8.84. C_28_H_22_N_4_S_2_O_3_BCuCl requires C, 52.99; H, 3.47; N, 8.83%). IR/cm^−1^ (Nujol mull): 1598_vs_ (C=N), 1419 (B−O), 2400 (B−H), 391 (Cu−O), 310 (Cu−Cl).

### Synthesis of [C_28_H_22_N_4_S_2_O_3_BZnCl] (4)

This was synthesized by a procedure similar to that described for complex [C_28_H_22_N_4_S_2_O_3_BCuCl] (3). Yield 0.6 g (57%) mp 220 ± 2°C (found: C, 52.84; H, 3.47; N, 8.78. C_29_H_24_N_4_S_2_O_3_BZnCl requires C, 52.83; H 3.45; N, 8.80%). IR/Cm^−1^ (Nujol mull): 1596_vs_ (C=N), 1423 (B−O), 2394 (B−H), 400 (Zn−O), 321 (Zn−Cl). *δ*_H_ (300 MHz, DMSO, TMS) 7.55-6.45 (ArH), 7.94–7.97 (HC=N), 0.54 (B−H). *δ*_c_ 127-125 (ArC), 165 (HC=N), 115(C−N), 151(C−S).

### Synthesis of [C_32_H_34_N_4_S_2_O_3_BCuSn_2_Cl_5_] (5)

**Scheme 1:**
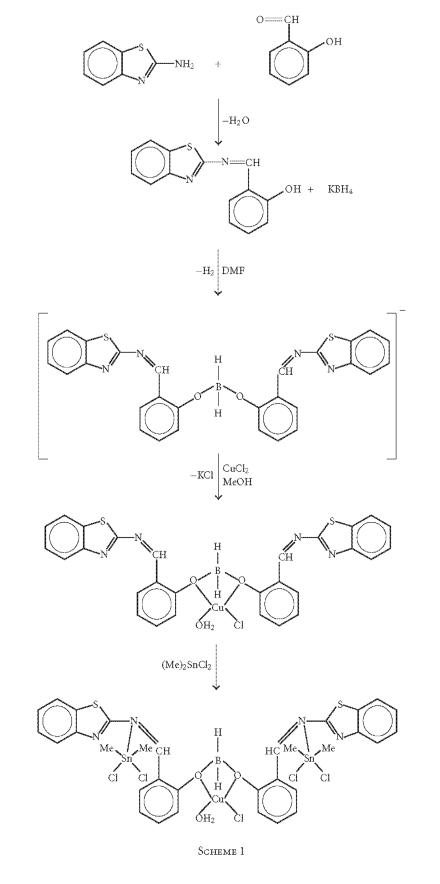


To a solution of C_28_H_22_N_4_S_2_O_3_BCuCl (0.634 g, 0.001 mol) in 40 mL DMF was added (CH_3_)_2_SnCl_2_ (0.438 g, 0.002 mol) in 1 : 2 molar ratio. The reaction mixture was refluxed for 48 hours on a water bath. A dark brown precipitate appears, which was isolated, filtered off, washed with hexane, and dried in vacuo over fused CaCl_2_. Yield 0.52 g (49%) mp (dec.) 340 ± 2°C (found: C, 35.84; H, 3.16; N, 5.20. C_32_H_38_N_4_S_2_O_7_BCuSn_2_Cl_5_ requires C, 35.82; H, 3.17; N, 5.22%). IR/Cm^−1^ (KBr): 1521 (C=N), 1420 (B−O), 2400 (B−H), 390 (Cu−O), 311 (Cu−Cl), 462 (Sn−Cl), 420 (Sn−N) 546 (Sn−C). (Scheme 1).

### Synthesis of [C_32_H_34_N_4_S_2_O_3_BZnSn_2_Cl_5_] (6)

This was synthesized by a procedure similar to that described for complex [C_32_H_34_N_4_S_2_O_3_BCuSn_2_Cl_5_] (5). Yield 0.50 g (59%) mp (dec.) 300 ± 3°C (found: C, 35.77; H, 3.16; N, 5.20. C_33_H_40_N_4_S_2_O_7_BZnSn_2_Cl_5_ requires C, 35.75; H, 3.16; N, 5.21%). IR/Cm^−1^ (Nujol mull): 1524 (C=N), 1421 (B−O), 2400 (B−H), 402 (Zn−O), 320 (Zn−Cl), 460 (Sn−Cl), 428 (Sn−N), 549 (Sn−C). *δ*_H_ (300 MHz, DMSO, TMS) 7.67-6.62 (ArH), 9.84-8.40 (HC=N), 0.55 (B−H), 1.21 (CH_3_). *δ*_c_ 130-124 (ArC), 168 (HC=N), 118 (C−N), 155 (C−S) 39.8 (Sn−C).

## RESULT AND DISCUSSION

The reaction of Schiff base (1) with KBH_4_ in 2 : 1 ratio yielded dihydro OO′ bis (salicylidene) 2,2′ aminobenzothiazolyl borate (2), which was utilized as a ligand for complexation with CuCl_2_ (3), ZnCl_2_ (4), and subsequent complexation of (3) and (4) with dimethyltindichloride yielded the bimetallic borate complexes (5) and (6), respectively.

All the complexes are air stable and are soluble in DMF, DMSO and are covalent in nature. The analytical data of the complexes conform to the structures proposed in Scheme 1.

### IR spectra

The IR spectrum of the ligand shows prominent stretching vibration at 2372–2400 cm^−1^ region due to *ν*(B−H). The BH stretch generally appears as a single peak in the regions 2400–2500 cm^−1^, but the presence of both ^10^B and ^11^B in natural boron results in the splitting of bands [22, 23]. Other characteristic frequencies due to the presence of the ligand appear at 1595 cm^−1^, 1449 cm^−1^, and 752 cm^−1^ assigned to *ν*(C=N), *ν*(C−N), and *ν*(C−S) vibrations, respectively [24–26]. The formation of borate is authenticated by the appearance of *ν*(B−O) band at 1391 cm^−1^ [27, 28], which is further confirmed by absence of *ν*(O−H) stretching vibration at 3422 cm^−1^ which was present in the Schiff base [29]. The *ν*(B−O) band, however, shifts to higher frequencies (28 cm^−1^) in the complexes indicating the participation of oxygen of borate in the formation of complexes.

In the far IR spectra of the monometallic complexes, sharp absorption bands appearing at 390–402 cm^−1^, 311–320 cm^−1^ are assigned to *ν*(M−O) and *ν*(M−Cl) vibration, respectively [30, 31]. The bimetallic complexes show absorption bands at 420–428, 546–549, and 460–462 cm^−1^ assigned to *ν*(Sn−N), *ν*(Sn−C), and *ν*(Sn−Cl), respectively, confirming the coordination of tin(IV) center to azomethine nitrogen and chlorine atoms [32, 33].

### Electronic absorption spectra

The electronic absorption spectra of the borate ligand and complexes reveal three strong bands in 40 000–25 000 cm^−1^ region which are attributed to intraligand and charge transfer transitions.

The complex (3) exhibits a broad and low energy band at 16 528 cm^−1^ which is attributed to d-d transition (^2^B_1*g*_ →^2^A_1*g*_), typical for Cu^II^ in square planar environment. The absorption spectrum of the (5) complex exhibits two MLCT bands at 34 013 cm^−1^ and 30 303 cm^−1^ and a broad band at 17 361 cm^−1^ attributed to d-d transition which is typical for copper(II) complex in square planar geometry [34]. Although there is a shift in the d-d absorption band of the (5) complex in comparison to the absorption band observed for the (3) complex which is attributed to the presence of Sn(IV) metal ion, the environment around the copper center does not alter much, it retains square planar geometry.

### EPR studies

The solid state X-band EPR spectrum of the Cu(II) complex recorded at LNT (77 K) was found to be anisotropic with only two peaks with “*g*” values *g*_⊥_ = 2.037, *g*_∥_ = 2.195, and *g*_av_ = 2.089, respectively. The parameter *g*_av_ was obtained according to the equation [(*g*_av_) = 1/3 (*g*_∥_ + 2*g*_⊥_)] and is in good agreement with corresponding anisotropy in square planar environment. The existence of *g*_∥_ > *g*_⊥_ suggested that the unpaired electron is localized in *d_x_^2^*−*y^2^* orbital of the Cu^II^ ion with “3*d* ^9^” configuration, that is, (*eg*)^4^, (*a*_1_ *g*)^2^ (*b*_2_ *g*)^2^ (*b*_1_ *g*)^1^, which is the characteristic of the square planar geometry [35].

The *g* values are related to the axial symmetry parameter *G* by the Hathaway [36, 37] expression *G* = (*g*_∥_ − 2)/(*g*_⊥_ − 2). According to Hathaway, if the value of *G* is greater than four, the exchange interaction is negligible, whereas when the value of *G* is less than four, a considerable exchange interaction is indicated in the complex. In the complex (5), the *G* value obtained was 5.27 which indicates that exchange interactions are absent.

### NMR studies

^1^H NMR spectra are particularly useful to confirm the formation of borate ligand. The absence of phenolic −OH resonance peak at 10.12 ppm in the ligand clearly indicates the formation of borate by the removal of hydrogen gas [38] and the presence of BH_2_ signal at 0.72 ppm [39]. The other signals due to N=CH proton and aromatic ring proton appear at 7.99-7.97 and 7.55-6.52 ppm, respectively. In the complexes (4) and (6), there is a slight upfield shift in BH_2_ signal due to the presence of metal ions in the proximity of BH_2_ protons. A new signal appears in the complex (6) at 1.21 ppm due to methyl protons of the dimethyltin moiety.

^13^C NMR spectra of ligand have been recorded in DMSO and carbon resonance signals appears at 129-124, 165, 153, and 114 ppm assigned to aromatic phenyl ring carbons, HC=N, C−S, and C−N groups, respectively. Upon complexation, there is a slight shift in aromatic ring carbon resonance due to the coordination of metal to the oxygen atom of phenyl ring. Furthermore, complex (6) registers a new signal at 39.8 ppm attributed to −Sn−CH_3_ carbons due to which CH=N carbon resonance gets altered slightly, this is also an indication of coordination of azomethine nitrogen to the diorganotindichloride. Other carbon signals remain unaltered in the complexes.

### Electrochemical properties

**Figure 1:**
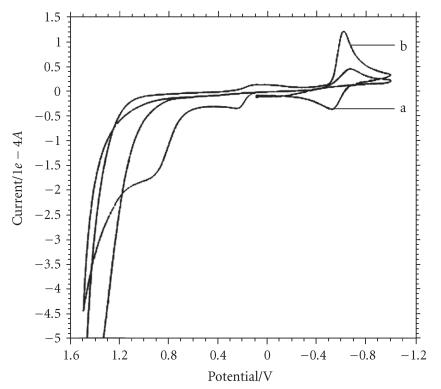
Cyclic voltammograms of the complex C_32_H_34_N_4_S_2_O_3_- BCuSn_2_Cl_5_ (5) in (a) the absence and (b) the presence of guanine in DMSO/buffer (5 : 95) at a scan rate of 0.1 Vs^−1^. Init E(V) = 0.1, high E(V) = 1.5, low E(V) = -1, Init P/N = P, scan rate (V/s) = 0.1, segment = 3, Smpl interval (V) = 0.001, quiet time (s) = 10, sensitivity (A/V) = 5*e* − 5.

The electrochemical behavior of the complex (5) has been examined by cyclic voltammetry to study the metallointeraction. The cyclic voltammogram of the complex (5) in absence of guanine, adenine, and calf thymus DNA was recorded in DMSO/H_2_O (5 : 95) at a scan rate of 0.1 Vs^−1^ in the potential range −1.2 to 1.6 V versus Ag/AgCl electrode. It exhibits a well-defined quasireversible redox wave Cu^II^/Cu^I^ attributed to one electron transfer process with *E_p_* value at −0.615 V and −0.519 V (Figure 1, curve a). For this couple, the difference between the cathodic and anodic peak potentials Δ*E_p_* is of the order 96 mV, a somewhat large peak to peak separations in comparison to Nernstian value (59 mV) observed for one electron transfer couple. Large peak width for one electron couple Cu^II^→ Cu^I^ is fairly common observation and is due to the reorganization of the coordination sphere during the electron transfer process [40, 41]. The ratio of anodic to cathodic peak currents *I*_pa_/*I*_pc_ is less then the unity (0.3). The criteria for reversibility of the process is satisfied as on increasing the scan rate; the voltammogram does not show any significant change, and current is proportional to V^1/2^ [42].

The electrochemical behavior of the complex (5) in the presence of the guanine, adenine, and calf thymus DNA in DMSO/buffer, DMSO/H_2_O, and DMSO/Tris buffer (5 : 95), respectively, are presented in Table 1.

The CV trace of the complex (5) in the presence of guanine shows a dramatic change in electrode potential *E_p_* values, while the cathodic peak potential shifts to −0.669 V (in comparison to solution without guanine *E_p_* = − 0.615 V), a positive shift of −0.054 V is observed. However, the anodic peak disappear completely (Figure 1, curve b) indicative of strong binding of the complex to guanine base.

**Figure 2:**
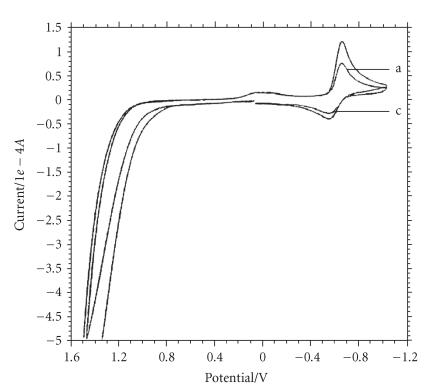
Cyclic voltammograms of the complex C_32_H_34_N_4_S_2_O_3_−BCuSn_2_Cl_5_ (5) in (a) the absence and (b) the presence of adenine in DMSO/H_2_O (5 : 95) at a scan rate of 0.1 Vs^−1^. Init E(V) = 0.1, high E(V) = 1.5, low E(V) = −1, Init P/N = P, scan rate (V/s) = 0.1, segment = 3, Smpl interval (V) = 0.001, quiet time (s) = 10, sensitivity (A/V) = 5*e* − 5.

The cyclic voltammogram of the complex (5) in the presence of adenine shows a slight shift in formal potential values (in comparison to the solution in absence of adenine) (Figure 2, curve c). Although it corresponds fairly well with quasireversible one electron redox couple, peak potential does not show any significant change (0.002 and 0.003 V). The CV trace of adenine bound complex clearly suggests that the binding of the complex (5) to adenine is possible but the degree of binding is much lower in comparison to guanine.

**Figure 3:**
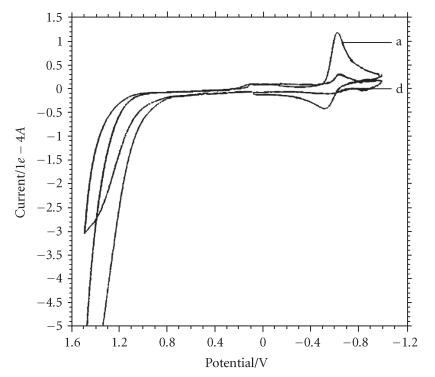
Cyclic voltammograms of the complex C_32_H_34_N_4_S_2_O_3_-BCuSn_2_Cl_5_ (5) in (a) the absence and (b) the presence of calf thymus DNA in DMSO/buffer (5 : 95) at a scan rate of 0.1 Vs^−1^. Init E(V) = 0.1, high E(V) = 1.5, low E(V) = -1, Init P/N = P, scan rate (V/s) = 0.1, segment = 3, Smpl interval (V) = 0.001, quiet time (s) = 10, sensitivity (A/V) = 5e − 5.

However, the cyclic voltammogram of the complex (5) in the presence of calf thymus DNA (Figure 3, curve d) shows a significant shift in electrode potential value; cathodic and anodic peak potentials both shift to positive values −0.642 V and −0.561 V, while for the solution of the complex (5) in the absence of calf thymus DNA, electrode potential values are −0.615, −0.561 V as depicted in Figure 1, curve a, indicating that both the copper(II) and copper(I) forms interact with the calf thymus DNA to the same extent and suggest strong binding with calf thymus DNA [43]. Employing a square redox scheme, the net shift in *E_1/2_* has been estimated from the ratio of equilibrium constants for the binding of Cu^II^ and Cu^I^ complexes to calf thymus DNA using the following equation:
(1)
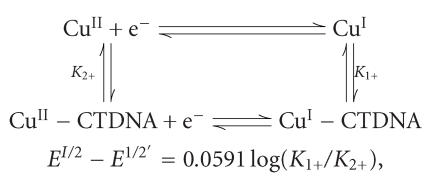

where *E* ^1/2^ and *E* ^1/2′^ are formal potentials of the Cu(II)/Cu(I) couple in the free and bound forms, respectively. The ratios of binding constants of *K*_2+_ and *K*_1+_ were corresponding to binding constants for the Cu(II)/Cu(I) species to DNA, respectively [44]. The ratio of binding constants of 1+ and 2+ species was less than 1 (0.670 for calf thymus DNA), which provides an evidence for the preferential stabilization of Cu(II) species.

### Kinetic studies

The interaction of the complex (5) with guanine, adenine, and calf thymus DNA in DMSO/buffer, DMSO/H_2_O, and DMSO/Tris buffer (5 : 95) was studied spectrophotometrically at 25°C under pseudo-first-order conditions.

**Figure 4:**
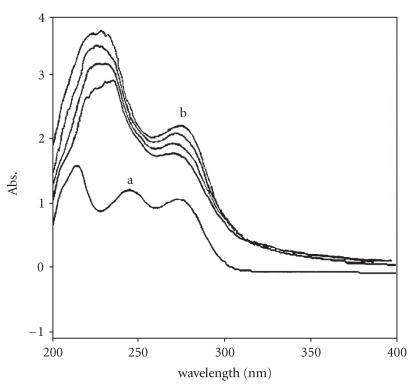
Absorption spectra of (a) guanine (1 × 10^−3^ M) dissolved in 9.2 pH buffer in the absence of the complex, (b) interaction of complex C_32_H_34_N_4_S_2_O_3_BCuSn_2_Cl_5_ (5) with increasing amount of guanine.

The electronic absorption spectrum of free guanine exhibits two characteristic bands at 244 nm and 273 nm. On addition of the complex (5), the UV band at 273 nm increased in intensity and shifted to 269 nm (a shift of 4 nm is observed), as shown in Figure 4. The binding of guanine with the complex (5) results in blue shift and increase in intensity which is attributed to “hyperchromism.” Hyperchromism was due to the breakage of intermolecular hydrogen bonds when bound to DNA and is consistent with many earlier reports for copper complexes [45, 46].

**Figure 5:**
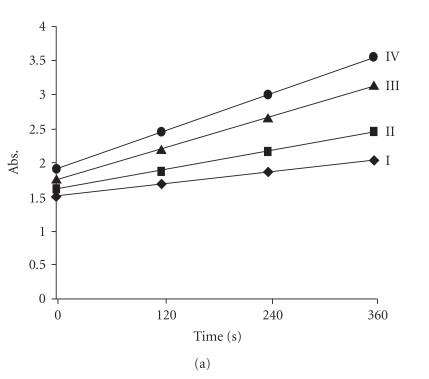

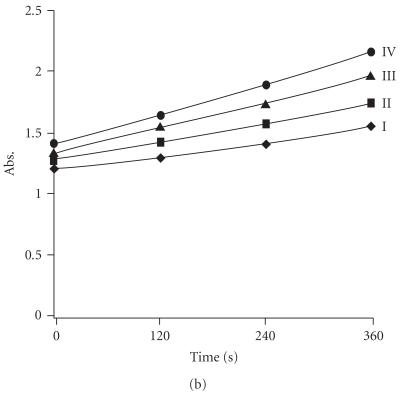

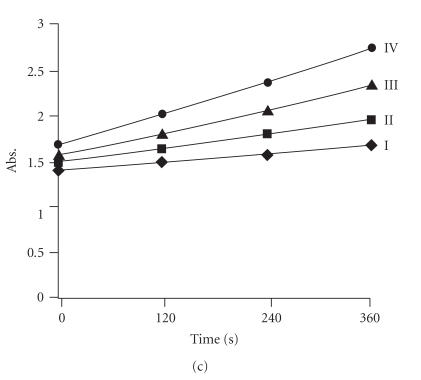
Plot of absorbance versus time at different concentrations of (a) guanine, (b) adenine, (c) calf thymus DNA.

**Figure 6:**
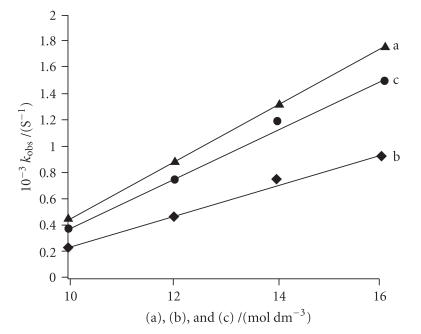
(a) Plot of *k*_obs_ versus guanine, (b) *k*_obs_ versus adenine, and (c) *k*_obs_ versus calf thymus DNA.

Kinetics of guanine binding to the complex (5) was studied at 269 nm (λ_max_ of the complex (5) + guanine) under pseudo-first-order condition keeping the concentration of complex constant (*c* = 1 × 10^−3^ M) and varying the concentration of guanine (*c* = 10 − 16 × 10^−3^ M) at different time intervals (Figure 5(a)). The rate constants *k*_obs_ were determined by the linear least squares regression method. An exponential log(*Aα* − *A_0_*) of absorbance against time plots gave a straight line indicative of pseudo-first-order reaction upto 80% completion of the reaction (Figure 6).

The electronic absorption spectrum of adenine shows a characteristic UV band at 260 nm. On addition of the complex (5), there is no significant shift in wavelength and a slight increase in absorbance. Although a slight hyperchromism is observed, but the degree of hyperchromism is insignificant in comparison to the binding of guanine to the complex (5), which shows relatively weak interaction with adenine base.

Kinetics of adenine binding to the complex (5) was carried out at 260 nm (λ_max_ of complex (5) + adenine) under pseudo-first-order conditions. Figure 5(b) shows time scan plot of interaction of adenine with complex (5) depicting a small change in absorbance intensity. The rate constant *k*_obs_ values were plotted by linear least squares regression method (Figure 6).

The interaction of the complex (5) with calf thymus DNA was carried out to obtain detailed information concerning the magnitude of the kinetic influence from the DNA environment as a function of position of guanine N_7_ within calf thymus DNA.

The interaction of the complex (5) to the calf thymus DNA was carried out at 260 nm (λ_max_ of calf thymus DNA) under pseudo-first-order conditions (Figure 5(c)). On addition of the complex (5) to the calf thymus DNA, absorption spectra reveal a sharp change in absorption intensity with a red shift of 3 nm. At different time intervals, the absorption maxima increases in intensity indicating “hyperchromic effect” with calf thymus DNA.

The kinetics is further studied by observed pseudo-first-order rate constants *k*_obs_ value, as they can be directly compared and used as a measure of the kinetic influence from surrounding DNA [47]. The observed rate constant 1.77 s^−1^ for guanine bound complex is of large magnitude in comparison to *k*_obs_ 1.52 s^−1^ value for calf thymus DNA bound complex. The rate constant for adenine bound complex is 0.94 s^−1^, which is much slow in comparison to guanine and calf thymus DNA. The complex shows preference for guanine over adenine due to two effects. When adenine binds, only a weak hydrogen bond is formed between the chloride ligand of the complex and H_2_N−C_6_ group of adenine; secondly, a significantly stronger molecular orbital interaction is identified in guanine in comparison to adenine. The presence of the electron withdrawing oxo group at the C_6_ position of the purine ring lowers the energy of the lone-pair orbital at N_7_ of purine base. The guanine molecular orbital has an energy of −6.877 eV, whereas −6.675 eV is obtained for adenine [48]. These studies have been demonstrated in a recent article of interaction of cisplatin with purine bases by Lippard et al [49]. Our investigations show that the complex C_32_H_34_N_4_S_2_O_3_BCuSn_2_Cl_5_ is strongly bound to calf thymus DNA via different modes. Cu(II) prefers to bind strongly to N_7_ of guanine base while the Sn^IV^ atom binds to the phosphate group [50]. Moreover, the affinity of Sn^IV^ with dinegative phosphate group is very strong due to its hard Lewis acidic nature. The binding to guanine is also kinetically preferred and supported by large *k*_obs_ value (1.77 s^−1^) for guanine bound complex.

Thus in conclusion, the complex (5) may first bind with phosphate group of calf thymus DNA, neutralize the negative charge of calf thymus DNA phosphate group, and cause contraction and conformation change of calf thymus DNA which is clearly evidenced by the „overall” hyperchromic effect observed in the absorption spectra.

## Figures and Tables

**Table 1:** Electrochemical data for complex (5) at a scan rate of 0.1 Vs^−1^ in the potential range −1.2 to 1.6 V.

System	*E_pc_*	*E_pa_*	*E*_1/2_	*I_pa_/I_pc_*	Δ*E_p_*

Complex (5) alone	−0.615 V	−0.519 V	−0.56 V	0.30	96 mV
Complex (5) + calf	−0.642 V	−0.561 V	−0.60 V	0.50	81 mV
thymus DNA					
Complex (5)	−0.669 V	—	—	—	—
+ guanine					
Complex (5)	−0.617 V	−0.516 V	−0.56 V	0.50	101 mV
+ adenine					
